# Supplementary material for: The Putative Auto-Inhibitory Domain of Durum Wheat Catalase (TdCAT1) Positively Regulates Bacteria Cells in Response to Different Stress Conditions
Source: Antioxidants (Basel). 2022 Sep 15;11(9):1820. doi: 10.3390/antiox11091820 (PMC9495866; doi:10.3390/antiox11091820)
Supplement: Supplementary file 1 [file antioxidants-11-01820-s001.zip › antioxidants-1890580-supplementary.pdf]

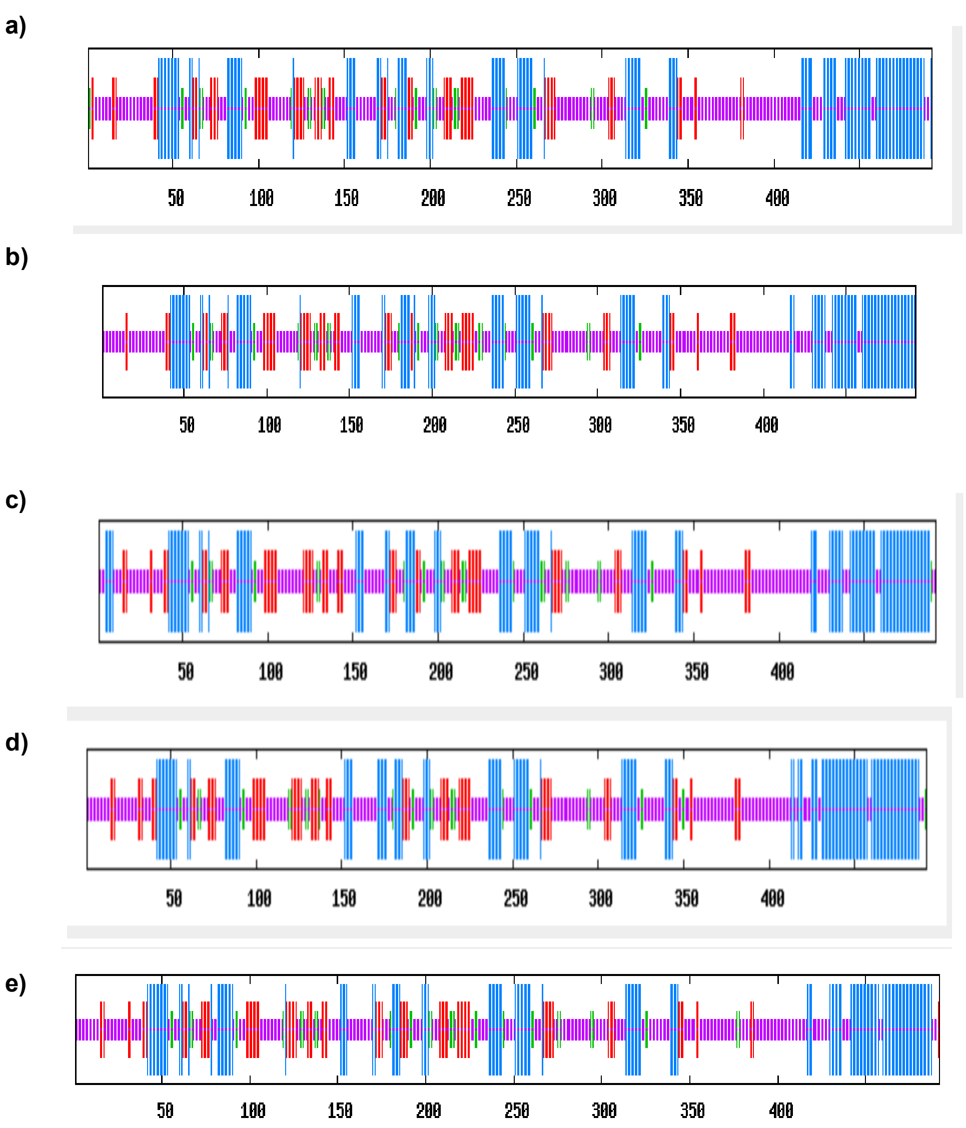

Supplementary Figure S1. Comparison of the secondary structures of CAT1s. **(a)** *Triticum durum,* **(b)** *Brachypodiumdictyosum* (XP_003558892.1), **(c)** alpine snowbell (*Soldanella alpine,* O24339*),* **(d)** upland cotton *(Gossypium hirsutum,* P17598*),* **(e)** *Aegilops taushii* (XP_020164896.1*)* and *Triticum aestivum* (Q43206.1*)* as revealed by SOPMA. The helix, sheet, turn and coil are indicated in vertical lines in order from the longest to the shortest.

a)

```
*
     MDPYKYRPSSSFNAPMWSTNSGAPVWNNDNSLTVESRGPILLEDYHLVEK   #    50
     IADFDRERIPERVVHARGASAKGFFEVTHDVSHLTCADFLRAPGVQTPVI   #   100
     VRFSTVIHERGSPETLRDPRGFAIKFYTREGNWDLVGNNFPVFFIRDGMK   #   150
     FPDMVHALKPNPKTHIQENWRILDLFSHHPESLHMFTFLFDDIGVPADYR   #   200
     HMDGSGVNTYTLVNRAGKAHYVKFHWKPTCGVKSLLEEEAVTVGGTNHSH   #   250
     ATKDLTDSIAAGNYPEWTFYIQTIDPDHEERFDFDPLDVTKTWPEDVVPL   #   300
     QPVGRLVLNRNIDNFFSENEQLAFCPGIIVPGVDYSDDKLLQTRIFSYSE   #   350
     TQRHRLGANYLLLPANAPKWSHHNNHYDGLMNFMHRDEEVDYFPSRFDPA   #   400
     KHAPRYPIPSRTLNGRREKMVIEKENNFKQPGERYRSMDPARQERFINRW   #   450
     IDALSDPRLTHEIKAIWLSCWSQADKSLGQKLASRLSSKPSM           #   500
%1   ...Y....SSS.......T...........S...................   #    50
%1   ...................S.......T..................T...   #   100
%1   ...S.......S..T...................................   #   150
%1   ...............................S..................   #   200
%1   ....S...............Y.......T....S................   #   250
%1   .T...T.............Y..T..................T........   #   300
%1   ................S.................Y.......T...SY..   #   350
%1   T...................S....................Y........   #   400
%1   .....Y............................Y.S.............   #   450
%1   ....S....T........S..S....S......S..SS....
```

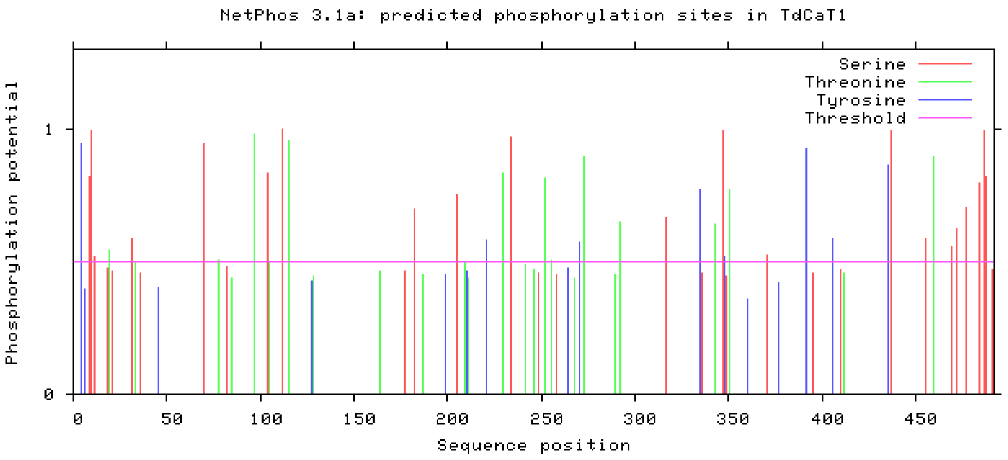

b)

Predicted Sites

Position	Peptide	Score	Cutoff	Cluster
86	HDVSHLTCADFLRAP	2,011	0	Cluster B
230	KFHWKPTCGVKSLLE	19,226	0	Cluster C
325	ENEQLAFCPGIIVPG	1,163	0	Cluster B
470	IKAIWLSCWSQADKS	0,322	0	Cluster A

Supplementary Figure S2. Bio-informatics analysis of TdCAT1 protein. **(a)** Identification of phosphorylation sites in TdCAT1 protein using NetPhos database **(b)** Identification of Nitrosylation sites in TdCAT1 structure using GPS-NSO software.

Supplementary Table S1. List of primers used in PCR amplification of TdCAT1 and its truncated forms. The forward primer CF was used for the amplification of all forms with the use of reverse primers corresponding of each one.

Amplified fragments	Nucleotide sequences	Primers names
**TdCAT1**	TCGAATTCATGGACCCCTACAAGTA	CF
**TdCAT$_{200}$**	ATCTCGAGTTACATGCTCGGCTTGG	CR1
**TdCAT$_{295}$**	ATCTCGAGGGCGGTAGTCGGCGGG	CR2
**TdCAT$_{340}$**	ATCTCGAGCCTCGGGCCACGTCTTG	CR3
**TdCAT$_{400}$**	ATCTCGAGGGTGAGGCGGGGGTCC	CR4
**TdCAT$_{460}$**	ATCTCGAGGGGTGAGGCGGGGGTC	CR5

Supplementary Table S2. Secondary structure analysis of plant catalase using SOPMA program (https://npsa-prabi.ibcp.fr/cgi-bin/npsa_automat.pl?page=/NPSA/npsa_sopma.html). 14 different catalase proteins were used to study the secondary structure of those proteins: [*Triticum turgidum*subsp durum]: TdCAT1 (GenBank: AKC00864.1); [*Triticum aestivum*]: TaCAT1 (GenBank: XP_044371531.1); [*Aegilops tauschii* subsp. *strangulata*] (GenBank: XP_020164896.1); [*Triticum monococcum*]: TmCAT1 (GenBank: QBZ38484.1); [*Brachypodiumdistachyon*]: BdCAT1 (GenBank: XP_003558892.1); [*Triticum dicoccoides*] (GenBank: XP_037426584.1); [*Panax ginseng*]: (GenBank: EU327037); [*Soldanellaalpina*)]: (GenBank: O24339); [*Gossypium hirsutum*] (GenBank: XP_016687939.1); [*Lotus japonicus*] (GenBank: AAR84578); [*Gossypium raimondii*] (GenBank: XP_012446280.1) and [*Arachis ipaensis*] (GenBank: XP_016167161.1).

**Protein**	α-helices	β-turns	random coils	extended strands
**TdCat1**	135	27	256	74
**TaCat1**	140	27	242	83
**AetCAT1**	127	30	258	77
**TmCAT1**	137	30	249	76
**BdCat1**	134	29	257	72
**AtCat1**	126	31	260	75
**TdcCAT1**	131	28	258	75
**PgCAT1**	138	29	250	75
**SaCAT1**	135	27	253	77
**GhCAT1**	139	29	249	75
**LjCAT1**	130	25	262	75
**EsCAT1**	137	28	255	72
**GrCAT1**	141	29	248	74
**AiCAT1**	134	32	247	79
